# The Perspective of Psychosomatic Medicine on the Effect of Religion on the Mind–Body Relationship in Japan

**DOI:** 10.1007/s10943-012-9586-9

**Published:** 2012-03-21

**Authors:** Mutsuhiro Nakao, Chisin Ohara

**Affiliations:** 1 Division of Psychosomatic Medicine, Teikyo University Hospital, Teikyo University School of Medicine, 2-11-1, Kaga, Itabashi, Tokyo, 173-8605 Japan; 2 Teikyo Graduate School of Public Health, Tokyo, Japan

**Keywords:** Buddhism, Japan, Psychosomatic medicine, Religion, Shintoism

## Abstract

Shintoism, Buddhism, and Qi, which advocate the unity of mind and body, have contributed to the Japanese philosophy of life. The practice of psychosomatic medicine emphasizes the connection between mind and body and combines the psychotherapies (directed at the mind) and relaxation techniques (directed at the body), to achieve stress management. Participation in religious activities such as preaching, praying, meditating, and practicing Zen can also elicit relaxation responses. Thus, it is time for traditional religions to play an active role in helping those seeking psychological stability after the Great East Japan Earthquake and the ongoing crisis related to the nuclear accident in Fukushima, Japan, to maintain a healthy mind–body relationship.

## Introduction

The Great East Japan Earthquake on March 11, 2011 (Normile 2011) resulted in more than 15,000 deaths and 4,000 missing persons. The crises related to the earthquake, tsunami, and nuclear accident in Fukushima Prefecture have inflicted great damage on the socioeconomic activities of Japan. From a medical perspective, effective strategies are needed to prevent epidemics of physical illnesses, such as cardiovascular diseases, and mental illnesses, such as post-traumatic stress disorder (PTSD) and depression, after these nationalwide disasters (Kario et al. 2011).

Facing death is among the most stressful events for individuals. Grief can lead to experiences in which feelings of distress combine with confused thoughts (Klass and Goss 1999). Although many experts assume that bereaved individuals eventually recover their equilibrium as a result of mourning processes, practitioners of psychosomatic medicine often see patients who experience extreme states that cause clinical impairment in their ability to function (Nakao et al. 2005). Grief care is important for maintaining a healthy mind and body, but Japanese individuals are said to have a relatively high threshold for visiting psychiatrists and to lack socially sanctioned customs for consulting psychologists about anxiety related to death and the problems of daily life (Nakao et al. 2008).

Under these circumstances, Japanese religionists offer at least two advantages that are potentially helpful to people suffering from mind–body distress. First, they have recourse to a body of thought that enables them to talk about life and death in plain language that is understandable to the general public (Ohta 2006; Tanaka 2010). As noted by studies of psychotherapy, modification of distorted cognitions and dysfunctional behaviors involves aspects of stress management (Nakao et al. 2012). Second, religionists have been trained to treat their own and others’ mind–body difficulties by using techniques including meditation and Zen practices. Such techniques can facilitate relaxation in the service of self-care in various diseases including hypertension, insomnia, and somatic symptoms with unknown etiologies (Benson 1975; Nakao et al. 2001b).

The purpose of this article is to explain the Japanese religious constructs that are related to health and to draw on our clinical and research experiences at clinics for psychosomatic medicine to discuss the ways in which religion may contribute to mind–body health.

## Definitions

In Western countries, religion is defined as an organized system of beliefs, practices, rituals, and symbols designed to facilitate closeness to a sacred or transcendent-like God, higher power, or ultimate truth/reality (Koenig et al. 2001; Moreira-Almeida et al. 2006). Religion usually includes specific beliefs rooted in an established tradition that arises in the context of a group of people holding common beliefs about life after death and the rules of conduct that should guide social behavior (Koenig 2009). According to the most recent national survey (Japanese Institute of Statistical Mathematics 2008), 73% of Japanese individuals do not believe in religion. This figure seems anomalous in light of reports that the total number of believers in all religions was larger than the total population (i.e., 120 million) of Japan (Japanese Agency for Cultural Affairs 2011) (Table 1). Thus, the finding that 73% of Japanese individuals do not believe in religion does not necessarily mean that most Japanese are non-religious. Rather, it may mean that the ancient faiths of Shinto or Shintoism and the newly introduced religion of Buddhism have been integrated (the syncretization of Shintoism and Buddhism or *Shin*-*Butsu* *Shugo* in Japanese) during the long 2,000+-year history of religion and have eventually become so deeply incorporated into the psyches of Japanese individuals that they are no longer recognized as religions. This notion is discussed in detail in the next section on the historical background of these issues.

**Table 1:** The number of registered religious groups, instructors, and fellows in Japan: results from the 2009 statistical survey of religion (Japanese agency for cultural affairs)

	Groups	Instructors	Fellows
Shintoism	88,812	76,478	108,427,100
Buddhism	85,802	344,044	87,506,504
Christianity	9,275	39,443	2,369,484
Other	38,834	217,180	8,880,135
Total	222,723	677,146	207,183,223

A psychosomatic illness is defined as any physical condition with organic or functional damage that is affected by psychosocial factors during the process of its onset or development (The Japanese Society of Psychosomatic Medicine 1991). This definition corresponds largely to that presented in the Diagnostic and Statistical Manual of Mental Disorders, Third Edition, Revised and Fourth Edition published by the American Psychiatric Association: “psychosocial factors affecting general medical conditions (316.00)” (American Psychiatric Association 2000). In Japan, seven universities and a few regional hospitals have separate and independent psychosomatic medicine departments, and almost 3,000 physicians and psychotherapists are members of the Japanese Society of Psychosomatic Medicine (Nakao et al. 1998). Patients with psychosomatic illnesses and those with specific diagnoses such as chronic pain, insomnia, and cardiac conditions are often referred to psychosomatic clinics.

## Historical Background of Japanese Religions

Table 2 summarizes the Japanese history related to religion. “Japan,” *Nihon* in Japanese, refers to the origin of the sun. This appellation originated in the middle of the sixth century, when the government formally decided to introduce Buddhism from Kudara, a country in the western part of the Korean Peninsula (Shimizu 1997). This action was aimed at transferring the culture from East Asia, developing the Japanese political system and demonstrating the independence of Japan to the Chinese Emperor Zui (A.D. 581–618).

**Table 2:** History of religion-related issues in Japan

Years	Japanese era	Religion-related issues
145th–10th centuries BC	Jomon (hunter-centered life)	Belief in the spirituality of nature including trees, thunder, and animals
9th century BC to 3rd century AD	Yayoi (development of rice growing)	Development of Shamanism; formation of the concept of *Kami* and the original faith of Shintoism with an emphasis on beliefs about sun and water
3rd–6th centuries	Kofun (process of a united nation)	Establishment of Shintoism; belief in the spirituality of regional chiefs and the development of worship of the emperor as a family of *Kami*
6th–8th centuries	Asuka (administration under the legal codes)	Official introduction of Buddhism from East Asia; building of *Ho*-*ryu*-*ji*, a temple that is the oldest wooden structure in the world, in 607
Years 710–794	Nara (43rd–50th Emperors)	Nara’s Great Statue of Buddha was built in the *To*-*dai*-*ji* temple in 752
8th–12th centuries	Heian (51st–82nd Emperors)	Esoteric Buddhism; *Tendai*-*shu* and *Shingon*-*shu* were translated in around 800, which accelerated the syncretization of Shintoism and Buddhism
12th–14th centuries	Kamakura (Shogunate or warrior government in a suburb of Tokyo)	New Buddhism such as Jyodo-shu, Nichiren-shu, and Zen-shu spread throughout the general public and warriors. Kamakura’s Great Statue of Buddha was built in the *Kotoku*-*in* temple in 1243
14th–16th centuries	Muromachi (Shogunate or warrior government in Kyoto)	Zen-shu became more common in the warrior culture, and *Jodo*-*kyou*-*kei* became common in the general public
15th–16th centuries^a^	Sengoku (age of civil wars around the country)	Christianity was first introduced by Francisco de Xavier in 1549; it was affected by several feudal lords in warring states
Years 1573–1603	Azuchi-momoyama (Samurai Leadership by Nobunaga Oda and Hideyoshi Toyotomi)	During the process of the re-unification of the nation, Oda and Toyotomo attempted to separate government and religion; Oda suppressed a Buddhist uprising, and Toyotomi ordered the expulsion of Christian priests in 1587
Years 1603–1868	Edo (Shogunate or warrior government by Tokugawa in Tokyo)	Through a policy of national isolation, Christianity was suppressed and Confucian Shintoism was developed with the support of the teachings of Chu Hsi Confucianism
Years 1868–1912	Meiji (restoration of Imperial Rule, 122nd Emperor)	Meiji government ordered a separation of Shintoism and Buddhism in 1868 and proposed a state endorsement of Shintoism to complete a thorough elimination of imperialism
Years 1912–1926	Taisyo (123rd Emperor)	World War I occurred in 1914, and the maintenance of the public order act was established in 1925 to restrict ideology
Years 1926–1989	Showa (124th Emperor)	After the end of World War II in 1945, freedom of ideology was ensured by the Japanese Constitution
Years 1989–present	Heisei (the present Emperor)	Sarin attack on Tokyo’s subways by the Aum Shinrikyou religious group in 1995. Occurrence of the Han-Shin Awaji Earthquake in 1995, the Mid-Niigata Prefecture Earthquake in 2004, and the Great East Japan Earthquake in 2011

^a^The Sengoku Era overlaps with the Muromachi Era. Although the Muromachi Shogunates appear to have lasted until 1573, when Oda exiled Shogun Asikaga, their power had already been lost after the Onin War (1467–1477). Thus, most historians place the *Sengoku* (meaning wars among countries) era between the Muromachi and Azuchi-momoyama eras

The original faith, Shintoism, preexisted the introduction of Buddhism. The birth and development of Shintoism is regarded as closely associated with the lifestyle change involved in moving from a hunting-centered to a rice-growing era. With the development of farming technology, people were able to remain geographically stable but prepared for and prayed about natural disasters such as drought, flooding, and temperature fluctuations. Thus, Shintoism held that a divine spirit dwelled in all of nature and brought joy and bounty to life (Ohta 2006; Hansen 2007). The “Sun Goddess” (*Amaterasu Oomikami* in Japanese) is the greatest among all the gods and goddesses (*Kami* in Japanese), according to the oldest chronicles of Japan (*Nihon*-*shoki* in Japanese). The Shinto concept of *Kami* differs completely from the Christian concept of God (Ohta 2006). It is assumed that the Sun Goddess is both the principal deity of nature and the ancestor of the Japanese Imperial Family, which has an unbroken line of descent from the first (Jinmu) or the twenty-sixth (Keitai) emperor to the one hundred and twenty-fifth emperor (the present Emperor), a period spanning 1,600–2,000 years or more (Shimizu 1997).

It took approximately 200 years to thoroughly syncretize Shintoism and Buddhism and integrate the resulting body of thought into daily life. The symbolic syncretization of Shintoism and Buddhism is exemplified by the presence of a gateway to a Shinto shrine (*Torii* in Japanese) in a temple and by the offering a prayer to Buddha in a shrine. This flexibility may also be reflected in the attitudes of modern Japanese individuals who, for example, celebrate Christmas on December 25 (Christianity), listen to a temple bell toll 108 times on December 31 (Buddhism), and watch the sun rise at the seashore or on a mountain on January 1 (Shintoism). Foreigners also seem to find it strange that a Japanese bride wears a Western wedding dress immediately after participating in marriage vows in front of Shinto’s *Kami* wearing special Japanese clothes (*Kimono* in Japanese) and a wig adorned with white cloth (*Tsuno*-*kakushi* in Japanese).

The third factor affecting the origin of religious spirituality among Japanese individuals is ancient Chinese ideology, including Confucianism and Taoism (Shimode 1987). The introduction of this ideology was not as abrupt as that of Buddhism, and Chinese ideology was gradually integrated into the original Shinto beliefs and was thereby included in the syncretization of Shintoism and Buddhism that occurred through the cultural exchanges of people during the fifth to the eighth centuries. The essence of this ideology is “Qi,” life or cosmic energy. As implied in Tables 1 and 2, neither religionists nor the leaders of Confucianism and Taoism were established in Japan. Indeed, it is only the ideology of the two religions to which common Japanese people subscribe. Consider, for example, the famous Japanese proverb that holds that illness is caused by Qi. The Japanese word for disease, *byo*-*ki,* was formed by the two Chinese letters, *byo* (illness) and *ki* (Qi).

In modern Japanese history, the authority of the Emperor and therefore of Shintoism was emphasized from the middle of nineteenth century to the end of World War II in 1945. This period was the age of imperialism, occurring after the power of the shogunate or warrior (*Samurai* in Japanese) government had collapsed. After World War II, religious liberty was guaranteed by the Constitution of Japan, and a variety of religions, including Christianity, independent religious organizations, and other religions spread throughout the country.

## Cultural Background Related to Japanese Religions

Although the three belief systems, that is, the original faith of Shintoism, the Asia-oriented faith of Buddhism, and the ideology of Qi, are theoretically separate, they have been regarded as identical for more than 1,000 years with respect to the Japanese sensibility about life (Shimode 1987). The Westernized era of imperialism and democracy lasted for less than 150 years. Thus, the concept of mind–body unity, which the three beliefs have consistently advocated, comprises a part of the foundation of Japanese thinking.

Defilement and purification have key roles in Shinto spirituality (Ohta 2006; Hansen 2007), as reflected in customs of daily bathing and ceremonies to ward off bad luck (*Oharari* in Japanese) held at Shrines. The purification of one’s body simultaneously purifies one’s mind and one’s immediate environment. Similarly, if the environment surrounding someone or his/her mind is defiled, his/her body is simultaneously defiled. In this sense, the three dimensions of mind, body, and external reality are treated as identical, and one domain is never privileged over the others.

The Buddhist principle of reincarnation emphasizes that all things are engaged in the endless circle of birth. It holds that all joys, sorrows, and pains are relieved by entry into Nirvanaattain Buddhahood, supreme enlightenment. In Japan, Buddhist ideas gradually changed as they became syncretized with Shintoism and grew to be associated with the abhorrence of living in this impure world versus in the Pure Land (*Enri*-*edo* in Japanese). According to this concept, the real world is full of defilement and must be denied; however, such denial is not limited to body or soul. Indeed, the existence of the human body, including the mind, as well as of the world in which the human body lives is totally denied. Thus, the mind and body are not regarded as separate in Japanese Buddhism. According to Shintoism, high mountains are spiritual places in which the *Kami* exist. In some kinds of Buddhism (*Shugen*-*do* in Japanese), the physical burdens of walking and climbing long distances in such mountains are important forms of training for the emancipation from worldly attachments. Later, in the shogunate, warriors often performed Zen practices and meditation to enhance their minds/bodies.

According to Taoism, Qi circulates in the human body and is the fundamental element of all things in nature or the universe. Taoism does not distinguish between the mind and body of humans or between the energies inside and outside the body. When the flow of Qi is obstructed in a human body, the life force and vigor of an individual are considered lost, and illness or death is thought to occur as a result. The Japanese common greeting *Genki*-*desuka*? (“Are you fine?”) inquires about whether the recipient has restored his/her Qi. Additionally, *Kimochi*-*ga*-*warui* (“I do not feel well”) means that something is wrong with one’s Qi. This concept is also expressed in domains other than health. For example, *Ten*-*ki* (weather) literally means the Qi in the sky, *Kei*-*ki* (economy) literally means a view of Qi, and *Ke*-*hai* (sign or indication) literally means a glimpse of Qi. In the absence of the word Qi, Japanese individuals cannot converse. Moreover, the ideologies of Qi, Shintoism, and Buddhism constitute the fundamental unconscious thoughts of Japanese individuals.

## Mind–Body Connections in Psychosomatic Illness

In terms of psychopathological mechanisms, alexithymia is known to be associated with the onset and development of psychosomatic illnesses (Sifneos 1973). Alexithymia, a personality construct derived from clinical observations of patients with psychosomatic illnesses, is characterized by difficulty distinguishing between emotions and bodily sensations. The Toronto Alexithymia Scale (TAS) and its modified versions, the TAS-R and TAS-20 (Taylor et al. 1992; Bagby et al. 1994), are among the most common instruments used to measure this construct. Evidence has suggested that the tendency to develop functional somatic symptoms is associated with alexithymia. Our recent study found that the degree of somatosensory amplification was significantly correlated with that part of the TAS-20 that addresses difficulty in identifying and describing emotions among outpatients with psychosomatic illnesses (Nakao et al. 2002). It makes sense that somatosensory amplification would be statistically and clinically linked to alexithymic characteristics.

Additionally, although depression itself does not meet the criteria for a psychosomatic illness, somatic symptoms are common in depression (Nakao et al. 2001c). In some cases, including so-called masked depression or mild depression, the affective and cognitive symptoms of depression are hidden behind a variety of somatic complaints. More than 30,000 people (25 per 100,000) have committed suicide in Japan each year during the last 15 years (Nakao and Takeuchi 2006). The close association between depression and suicide has been widely accepted. From a risk-management perspective, it is clinically and economically important to detect depression early because earlier detection reduces the direct costs of prolonged depression, including the costs of medications, hospital care, and community-based services; it also reduces indirect costs such as lost earnings, lost productivity, and unemployment. In our previous studies of white-collar workers, the prevalence of major depression was significantly associated with the total number of somatic symptoms, and the area under the receiver-operator-characteristic (ROC) curve was 0.92 for men and 0.81 for women, reflecting the sensitivity and specificity of the total number of somatic symptoms for detecting major depression (Nakao and Yano 2003). Somatic symptoms of low back pain, dizziness, and abdominal pain were significant risk factors for developing major depression during the subsequent year (Nakao and Yano 2006). The findings of these studies identified complaints of somatic symptoms as a simple and useful predictor of major depression.

Although pharmacotherapy, such as antidepressants or anxiolytics, is usually the first line of defense in the treatment for psychosomatic illnesses (Nakao et al. 2007), psychotherapy, and relaxation technique are also frequently used in psychosomatic clinics (Nakao et al. 1998). Psychotherapy includes cognitive-restructuring, coping skills, stress management, and body awareness. In our previous studies of a 10-week stress-management program, outpatients with a variety of somatic symptoms were taught cognitive-restructuring skills; they learned to recognize the automatic thoughts and cognitive distortions associated with stress-related physical and psychological symptoms (Nakao et al. 2001a). They also attended lectures and participated in group discussions focused on self-control and coping (problem-, emotion-, and physical-focused coping styles). The lectures addressed using humor as a coping mechanism, empathetic communication and social support, and relapse-prevention strategies. Participation in a series of psychoeducational programs and physiological trainings in relaxation led to improved stress-management skills, which resulted in decreased psychological and somatic symptoms among patients. More specifically, the self-reported frequency of somatic symptoms, the degree of discomfort, and the extent to which symptoms interfered with daily activities were significantly reduced, and significant reductions in anxiety and other forms of psychological distress were also observed. High levels of pretreatment anxiety predicted a decrease in the total number of somatic symptoms reported, and these results were confirmed in another recent study including a randomized controlled trial (Nakao et al. 2011).

## Application of Japanese Religion to Psychosomatic Medicine

Psychosomatic medicine focuses on the connection between mind and body in the diagnosis of and treatment for medical illnesses. Importantly, because all Japanese traditional religions assume that mind and body comprise a unit that operates in harmony with nature, this has not been an area of conflict among different belief systems in Japan (Shimode 1987; Ikemi 1996). Thus, cooperation between religionists and medical practitioners may benefit efforts to manage individuals with extant mind–body problems and enhance attempts to prevent such problems from arising (Mueller et al. 2001).

For example, a working group of religionists was established in 2007 to work on issues related to suicide. They organized a symposium for suicide prevention, arranged phone, and mail counseling for people with suicidal ideation, held memorial services for those who committed suicide, and conducted meetings for the bereaved family members of individuals who had committed suicide (Japanese Working Group of Religionists for Suicide Problems 2011). The management of bereaved persons is a particularly suitable role for religionists because they frequently address issues related to death as part of their professional responsibilities. In our previous study (Nakao et al. 2005), the Texas Inventory of Grief (Faschingbauer et al. 1977) was administered to Japanese women experiencing the death of a spouse or a first-degree relative, and scores on the inventory were significantly correlated with those on the first TAS-20 factor, “difficulty identifying feelings,” and on the tension–anxiety and depression scales of the Profile of Mood States (McNair et al. 1971). Grief reactions due to bereavement are closely associated with alexithymic characteristics and mood states; thus, the involvement of religionists in efforts to promote communication with those who wish to commit suicide and with bereaved family members may help these individuals to express their emotions and regulate their mental status.

Relaxation techniques are often taught by religionists, especially Buddhists. These include a simple prayer to Amida Buddha say, mindfulness meditation, Zen meditation in a cross-legged position, and Qigong or breathing exercises. According to the Cochrane Database of Systematic Reviews (Dickinson et al. 2008), the effects of a variety of relaxation therapies on cardiovascular outcomes and blood pressure were evaluated in people with elevated blood pressure. A meta-analysis indicated that relaxation resulted in small, but statistically significant reductions in systolic blood pressure (mean difference: −5.5 mmHg, 95% confidence intervals CI: −8.2 to −2.8) and diastolic blood pressure (mean difference: −3.5 mmHg, 95% CI: −5.3 to −1.6) in the experimental compared with the control group. More specifically, techniques of progressive muscle relaxation, cognitive–behavioral therapy, and biofeedback were associated with statistically significant reductions in the blood pressure of hypertensive patients. Biofeedback is a type of relaxation training and is part of a group of non-pharmacological therapeutic procedures that use electronic instruments to measure, process, and provide information to patients regarding their neuromuscular and autonomic nervous system activity in the form of analog (or binary) and visual (or auditory) signals. According to the results of our previous meta-analysis (Nakao et al. 2003) of a total of 22 randomized controlled studies with 905 patients with essential hypertension, reductions in systolic and diastolic blood pressure were significantly greater among those receiving the biofeedback intervention (by 7.3 (95% CI: 2.6–12.0) and 5.8 (95% CI: 2.9–8.6) mmHg) compared with those who visited clinics or self-monitored blood pressure. In the context of recent technical improvements in physiological monitoring and data processing, it may be possible for religionists to conduct relaxation training such as meditation by relying on easy-to-use systems for monitoring physiological data as well as on conventional psychological questionnaires.

Psychosomatic specialists and religionists are faced with three problems when dealing with psychosomatic conditions. As previously discussed, some alexithymic patients visit clinics for somatic symptoms instead of for psychological distress. Such individuals find it difficult to identify and express emotions, and these patients can be diagnosed and managed by psychosomatic physicians and other medical specialists. Second, some people continue to work in situations involving psychological pressure despite somatic dysfunctions. In other words, these individuals also find it difficult to identify and express somatic conditions; they will never visit a clinic or independently change their lifestyles before somatic dysfunction leads to severe illness. These individuals can be managed by both medical specialists and religionists. Third, beyond the connection between mind and body, some people find it difficult to identify and express gratitude about the benefits of nature (i.e., the feeling that we are alive and live in nature) (Ikemi 1996). These individuals can be managed only by religionists due to the need to discuss the meaning of life and death. Hospitals, terminal care facilities, and hospices often provide forums for such life/death discussions (Ando et al. 2010); the role of religionists in these facilities is also important.

## Conclusions

Although most Japanese do not acknowledge subscribing to a religion, they nonetheless acquire religious notions through their tradition and culture. The original faith of Shintoism, as well as Asia-oriented Buddhism and the ideology of Qi, adopts a shared approach to life, and a deep belief in mind–body unity underlies the fundamental thinking of Japanese individuals. Approaches to both the mind and the body are essential ingredients of efforts to use stress management to treat a variety of medical conditions; these approaches consist of psychotherapies (mind) and relaxation techniques (body). Japanese religionists are often skilled at teaching techniques related to both approaches (e.g., preaching, prayer, mindfulness, meditation, and breathing exercises). More than 220,000 religious groups are officially registered in Japan; these groups have more than 670,000 religious instructors (Table 1). The use of this network of religious groups to contribute to the maintenance of mind–body health and the prevention of stress-related diseases seems advisable as Japan faces the challenges resulting from accidents related to the Great East Japan Earthquake that occurred on March 11, 2011.
